# Feelings expressed by professionals caring for children and teenagers victims of sexual violence*

**DOI:** 10.1590/1518-8345.7157.4251

**Published:** 2024-08-19

**Authors:** Marimeire Morais da Conceição, Climene Laura de Camargo, Maria Carolina Ortiz Whitaker, Camila Tahis dos Santos Silva, Nildete Pereira Gomes, Lúcia Cristina Santos Rusmando

**Affiliations:** 1 Universidade Federal da Bahia, Salvador, BA, Brazil.; 2 Secretaria de Saúde do Estado da Bahia, Escola de Saúde Pública da Bahia Professor Jorge Novis, Salvador, BA, Brazil.; 3 Scholarship holder at the Fundação de Amparo à Pesquisa do Estado da Bahia (FAPESB), Brazil.; 4 Universidade Federal da Bahia, Escola de Enfermagem, Salvador, BA, Brazil.; 5 Scholarship holder at the Conselho Nacional de Desenvolvimento Científico e Tecnológico (CNPq), Brazil.; 6 Universidade Federal da Bahia, Maternidade Climério de Oliveira, Salvador, BA, Brazil.

**Keywords:** Patient Care Team, Domestic Violence, Child Abuse, Sexual, Hospital Care, Child, Adolescent

## Abstract

**Objective:**

to describe the feelings expressed by health professionals when caring for child and adolescent victims of sexual violence from the theoretical perspective of Symbolic Interactionism.

**Method:**

qualitative research carried out with 30 female health professionals. An instrument was used consisting of closed questions for sociodemographic data and a script with open questions for interviews. The data was organized and analyzed using Nvivo software version 12, according to Bardin’s proposal, from the perspective of Symbolic Interactionism in the work of Charles Morris. The project was approved by the Research Ethics Committee.

**Results:**

five thematic categories emerged, revealing feelings of empathy, fear, indignation, suffering, and consternation. These feelings remained in the interviewees’ memories, making caring for child and adolescent victims of sexual violence a moving and difficult experience that deeply marks the life of the health professional.

**Conclusion:**

there is a need to adopt strategies to support the mental health of professionals who work in services that provide general care to children and adolescents, considering that there is a possibility that they will provide care to child and adolescent victims of sexual violence in compliance with pre-existing public policies.

## Highlights

**(1)** Feelings stem from individual impressions in line with social interaction.

**(2)** Caring for child and adolescent victims has a negative impact on professionals’ emotions.

**(3)** Service managers need to pay attention to the health of professionals who assist victims.

## Introduction

Child and adolescent sexual violence (CASV) encompasses a multiplicity of sexual conduct with children and adolescents, whether consensual or not^1^ ^)-(^ ^2^. In Brazil, this concept has been classified into three categories: sexual abuse; commercial sexual exploitation and human trafficking^3^ ^)-(^ ^4^, which includes modalities propagated through the use of the Internet, such as sexting, sextortion and virtual rape^5^, which corroborates international positions on the subject^1^ ^),(^ ^5^. 

According to the United Nations (UN)^6^ and the World Health Organization (WHO)^5^, one billion children and adolescents in the world between the ages of two and 17 are victimized. A study reveals a prevalence of sexual violence of between 14.5% and 89.4% among 227 African adolescents^7^; similarly, in Brazil, the recent Epidemiological Bulletin warns that sexual violence is most often perpetrated against children aged between five and nine and adolescents aged between 12 and 14 who are female^8^. 

Despite these alarming figures, the invisibility of the cases is a fact. International studies point to the difficulty in identifying cases^9^ ^)-(^ ^12^. Consequently, it can be inferred that professional care for victims, even today, is a challenge made more difficult by socially imposed taboos in various cultural contexts. This situation is confirmed in a study carried out in Saudi Arabia with 300 Primary Health Care physicians who, although they are aware of child abuse, do not report the cases, which leads to underreporting in the country’s systems^13^. 

Studies point out that assistance to victims involves humanizing reception and preventing other problems, as well as psychosocial and health monitoring^14^ ^)-(^ ^17^, a process that has recently been improved in the country through the Child and Adolescent Statute (ECA, in its Portuguese acronym)^3^ ^)-(^ ^4^. Besides, there is the availability of technological devices for reporting, but their existence does not preclude the establishment of human interactions^4^ ^),(^ ^18^ ^)-(^ ^19^. 

It is known that in order to interact, individuals need to have contact (visual, auditory, tactile and/or verbal) with others in order to identify situations, people and objects that are the target of efforts, actions and interventions^20^. Therefore, care for victims and their families is mediated by feelings, which are considered to be the driving force behind actions whenever there is an attribution of meaning to this interaction^19^ ^)-(^ ^20^, the influences of which are marked by living together in society^20^ ^)-(^ ^21^. 

Caring for child and adolescent victims of sexual violence requires emotional preparation, as dealing with feelings during this care constitutes a challenge. A study reveals an impactful scenario perceived by professionals when assisting victimized children and adolescents who have genital lacerations, intense bleeding, health conditions that express serious clinical complications and may culminate in death^22^. These situations are striking for the human experience and can cause difficulties to the healthcare team.

This can be explained by the fact that the interventions required to care for victims need to be mediated by empathetic interactions that touch on these feelings^19^. On the other hand, the literature shows that professionals at a psychosocial care center express feelings such as impotence and frustration when caring for child victims of violence^23^, but there is still a knowledge gap regarding the feelings expressed by professionals who care for children and adolescents victims of sexual violence.

The relevance of this study lies in the possibility of contributing to improving the emotional preparation of professionals working in health services at any level of care. This provision helps to implement recommendations from the Ministry of Health so that these health workers are prepared and involved in caring for victims and their families^4^. However, it also serves to support occupational health policies in health services and, consequently, to extend the reach of these policies to the various contexts in which children and adolescents are cared for, such as schools, nurseries, Guardianship Councils, and Specialized Police Stations, among others, given that the feelings expressed may extend to people who interact and live together in the same community. Therefore, the aim of this study is to describe the feelings expressed by health professionals when caring for child and adolescent victims of sexual violence from the theoretical perspective of Symbolic Interactionism.

## Method

### Type of study

This is a qualitative study presented according to the Consolidated Criteria for Reporting Qualitative Research (COREQ) tool adapted for Brazil^24^, product of a master’s thesis presented in 2020 in a post-graduate program in the country. 

### Study location

The research took place in a public hospital in the northeast of Brazil which provides general, outpatient, clinical, surgical, and urgent/emergency care for all age groups and genders. The approach to the field came about through the first author’s work as a nurse at the hospital. The open (clinics) and closed (critical care units) care units for pediatric and adolescent patients were visited. The project was presented to the hospital board, to those responsible for each of the care units and potential participants at a prior meeting.

### Study subjects

To select the participants, all the health professionals found in the care units were approached individually and invited to take part in the study. Once they accepted, the inclusion criteria were checked: working for more than a year in the institution and having provided care to children and adolescents who had suffered sexual violence. The exclusion criteria were: being absent from the research service for any reason, and working in diagnostic support units.

There were no refusals or withdrawals by those invited to take part in the study. Thus, of the 36 professionals approached at random, only 31 met the inclusion criteria, but one interviewee was discarded due to insufficient information.

### Data collection

Data collection took place in June and July 2019, conducted by the master’s student together with four nursing students (all female) who participated individually in the interview in order to check compliance with the planned script. All the students were linked to a research group, duly qualified to collect qualitative data by an extension course attended in the same year.

Initially, a structured questionnaire and a semi-structured interview script previously created by the researcher were applied, which included the following question: how did you feel while assisting the victims? However, throughout the reports, the professionals also mentioned the reactions observed in other professionals during the process of assisting the victims. 

The in-depth interviews were pre-scheduled with the participants. The conversations, which took place only once during the professionals’ working hours (day and night) and lasted an average of 45 min, were audio-recorded. It is worth noting that thematic saturation of the data was identified with the 23^rd^ participant and confirmed with seven more interviews that showed similarities in the experiences shared, responded to the proposed objective, and did not add to the interpretation and analysis of the data.

### Data analysis

After collecting the data, the students transcribed it, but only one participant agreed to read and correct her transcribed interview. In view of this, the correction and textual validation of the other participants was carried out by the main researcher. The thematic content analysis technique suggested by Bardin^25^ was used in three stages: a) pre-analysis; b) exploration of the material; c) treatment of the results and inference.

In the pre-analysis stage, the interviews were transcribed, printed out and read in their entirety. Preliminary themes were then extracted which prompted mention of feelings expressed which were also present in the researchers’ observations, in the discussions after each interview and which were noted in the reflective diary entries. 

The second phase - exploring the material - began manually. A reflection group met weekly to analyze, discuss, reflect on, and share impressions and interpretations of the data. The transcribed texts were persistently re-read (each interview was read at least eight times by the main researcher and three times by the academics). In this process, in-depth readings were carried out at the same time as repeated listening (at least five times) to each interview, to allow the researchers to assimilate the texts. After this process, the texts were imported into NVivo software version 12, where they were organized and systematized into codes, or thematic nodes.

To proceed to the third stage - interpretation - the participants’ statements were grouped into codes based on convergent information, giving rise to a total of 23 expressions and terms that were considered sub-themes or sub-categories, grouped by convergent meaning into thematic categories^25^. The analysis process was validated through discussions with three nursing academics and three PhD researchers, as well as a private moment with five research participants who confirmed the results presented. The data analysis process is shown in Figure 1. 

**Figure 1:** Examples of expressions that gave rise to thematic categories from the perspective of Symbolic Interactionism. Salvador, BA, Brazil, 2019

Symbolic Object	Expressions shown in social interactions	Meaning	Signifier
Child/adolescent sexually assaulted	“*it could be their children*”	Pondering their possible suffering when experiencing a similar situation (having their child/adolescent raped) and weighing up the suffering that the other may be experiencing when having their daughter raped.	Empathy
	“*think of the woman’s suffering with her daughter in this state*”		
	“*I put myself in the woman’s shoes*”		
Action of seeking information to elucidate suspected sexual violence	“*afraid of asking too many questions and their mother complain*”	Possible reactions of the victims’ relatives to questions related to the suspicion of sexual violence.	Fear
	“*afraid to say anything suspicious ... family threatens*”		
The aggressor’s actions and reactions to children and adolescents sexual violence	*“indignant*”	Reaction to sexual aggression against children and adolescents is to reject the aggressor. Opinion converges on society’s need for insurgency and intolerance against this act and against those who don’t express the same sentiment.	Indignation
	“*I resented ... the mother didn’t show any anger*”		
Presentation of a complex clinical picture that requires physical and emotional effort on the part of professionals	“*the physician cried a lot* [...] *I felt terrible*”	A reaction that reflects the perception that the victim is also suffering, or even the result of the physical and emotional efforts made during the service.	Suffering
	“*as if my energy had been sucked out*”		
Long period of time after being seen	“*to this day, it stays in my mind* [...] *everyone is emotionally sad*”	A reaction of deep discouragement in the face of some mobilizing situation; this reaction can last regardless of the loss (and/or physical distancing) or even the emotional distancing from a social object, in these cases, the victim’s story.	Consternation
	“*it takes me a while to process these stories* [...] *you can’t help but feel mobilized and sad*”		

The analyses, inferences and interpretations recommended by Bardin^25^ are anchored in the philosophical foundations of Symbolic Interactionism (SI), found in Charles Morris’ “Mind, Self and Society”. The forerunner of this theory was the social scientist George Herbert Mead, whose ideas were systematized by his physician advisor Herbert Blumer. During the 19^th^ century, SI was incorporated into social psychology, leading to a theoretical framework consisting of three basic premises: 1) the way a person interprets facts and acts depends on the meaning attributed to them; 2) meaning is built up through interactive processes in society; and 3) over time, meanings can change^20^.

This theory focuses on social interactions, human responses and the influence that an individual’s immersion in society has on their stance towards the situations they experience^20^. Thus, five thematic categories were identified: empathy, fear, indignation, suffering and consternation.

### Ethical aspects

The project was approved by the Research Ethics Committee and complies with the Resolutions of the National Health Council (466/2012 and 510/2016). In this way, all the people who took part in the research, after reading and agreeing, signed the two copies of the Free and Informed Consent Form.

The interviews were conducted in private rooms located in the research institution, in the presence of the lead researcher and a nursing student, to guarantee the confidentiality and secrecy of the information provided. In order to preserve the anonymity of the participants, their names have been replaced by “P” (referring to a professional), followed by an Arabic number, according to the order in which the interviews were carried out: P1, P2, P3 ... P30.

## Results

Thirty health professionals took part, 33.3% of whom were nurses, 33.3% nursing technicians, 10.0% social workers, 10.0% physicians, 6.7% nursing assistants and 6.7% psychologists. All the participants reported being female, self-reported gender identity cis female, sexual orientation heterosexual, 80% of black race/color and aged between 25 and 65. Moreover, 73.3% had completed more than ten years of professional training, 70% had worked in the health sector for more than ten years and 40% had worked in the hospital where the study was carried on for more than ten years. 

After the analytical process, five thematic categories emerged that represent the feelings expressed by professionals and evidenced in the reports, namely: empathy, fear, indignation, suffering and consternation, as described below.

### Empathy

According to the participants’ reports, the feeling most evoked was empathy. Empathy means having the ability to put yourself in the other person’s shoes without necessarily going through the same experiences as them. Empathy, as mentioned above, is a relevant and indispensable feeling for care:

*Sexual aggression against a child or adolescent has a huge emotional impact. You immediately think that it could be your children* (P11, Nursing technician).

*Your heart races! We see the situation of this human being, this child/adolescent. I don’t know how someone can take a child, a baby, as has already happened. I ask myself: what is happening to human beings?* [...] (P26, Social worker)*.*

*Women are victims of rape on a massive scale. This affects our place as women, who are subjugated. We think about the suffering of this woman, mother, grandmother when she sees her daughter in this state* (P12, Social worker).

*I put myself very much in a woman’s shoes. For me, it would be the worst thing I could suffer in my life.* [...] (P13, Nurse).

### Fear

Fear was another feeling expressed by the professionals in the study, which occurred mainly during the collection of information about the case of sexual violence. This information is collected in health services based on the reports of those responsible for the victims, laboratory tests, imaging, and anamnesis. Fear also occurred explicitly during the act of communicating the diagnosis (or even the suspicion of it) to the victims’ relatives:

[...] *the area was bleeding and very open. We were afraid of asking too many questions and the mother complaining to the hospital management that we were asking too many questions, embarrassing her!* (P1, Nursing technician).

*We were afraid to say that there was a suspicion of sexual violence because the family often threatens the team* (P19, Nursing technician).

*When the other technician and I went to change his diaper and do intimate hygiene, we noticed that his anus was very dilated.* [...] *we were afraid to say anything about the suspicion and the father would react badly* (P10, Nursing technician).

### Indignation

The collaborators report indignation, outrage and despair when dealing with victims of sexual violence, whether they are children or adolescents. Their feelings are so strong that, in some cases, they rebel against the aggressor:

[...] *a crying, frightened one-year and a half-old boy. The physician found fissures, keloids, and bleeding in the rectum. The anesthesiologist and I were very indignant!* (P2, Nursing assistant)*.*

[...] *the relative showed her genitals, threatened her [the teenager] with death if she told her parents. It was absurd! I was rooting for the aggressor to die!* (P14, Nurse)*.*

[...] *she was an innocent girl, who didn’t even know what had happened to her. Her genitals were lacerated. What I resented most was the mother, who at no time showed any anger* (P9, Nursing technician).

*The anesthesiologist* [physician] *was indignant! The whole team was indignant!* (P8, Nursing technician).

### Suffering

The professionals interviewed reported that they felt so affected by cases of sexual violence against children and adolescents that several members of the team said that crying was the main expression of the emotion they experienced (a mixture of anger, sadness, rage, and empathy with the victim). The suffering of the victims also reflected and generated suffering in the professionals during their care:

*The child was about two years old.* [...] *He was injured from the genitals to the anus. The physician cried a lot while he was doing the surgery. I felt terrible!* (P9, Nursing technician)*.*

*The surgeon and I were very shocked, because it was an eight-month-old child who arrived in a very serious condition, with significant injuries to the anus and vagina. I was very shaken emotionally!* (P25, Doctor)*.*

[...] *these are difficult cases that involve our feelings. After the service, I feel drained, as if my energy has been sucked out of me* (P27, Psychologist)*.*

### Consternation

The image of the victims and memories of their stories are recurrently present in the professionals’ memories, arousing feelings of consternation. According to the participants, this happened even after they had spent a long time caring for these children and/or adolescents:

*It happened a couple of years ago and, to this day, it still sticks in my mind when that child arrived with a bleeding perianal region and went to the operating room* [...] *to this day, everyone is emotionally sad* (P1, Nursing technician)*.*

*I looked after a child who was less than a month old and died.* [...] *all I could think about at home was that. For a long time, whenever I was working, I would look at the bed and all I could think about was her* [...] *and everyone is very sad about this memory* (P23, Nursing assistant)*.*

*The mother brought the girl to the emergency room because she suspected that the aggressor was a family member.* [...] *when I deal with cases of sexual abuse of children and adolescents, it takes me a while to process these stories because you can’t help but feel very mobilized and sad after dealing with a case like this* (P12, Social worker)*.*

## Discussion

According to the participants’ reports, the most common feelings evoked were: empathy for the victims; fear of reporting the suspicion of sexual violence, of directing care towards this problem and of being retaliated against; indignation at the fact that occurred (sexual violence against children and adolescents) and at the unexpected inertia of the victim’s family; suffering in the face of the clinical cases and care situations experienced; consternation at the victim, the facts reported and the care provided.

These findings highlight the complexity of the individual involved in caring for sexually victimized children and adolescents. Although CASV has been established in society and this is evident in several studies^7^ ^-^ ^12^, its real-life occurrence still mobilizes the emotions of working professionals to the point where they bring up feelings that can lead them either to complete paralysis or to initiate necessary care actions, a process that depends on each person’s perception of the world. 

According to Morris, perception is a kind of mediator necessary for interactions between people^20^. From this perspective, the provision of care requires empathy, which makes it possible to create bonds of tenderness, understanding and affective reciprocity. This is because, by using empathy to provide care to child and adolescent victims of sexual violence, health professionals involve feelings of fraternity and compassion, which are considered important for human care^21^. Empathy is a feeling that gives an individual the psychological capacity to feel what another person would feel if they were in the same situation^19^ ^)-(^ ^21^ which occurs through human interactions.

National and international studies on the experience of domestic violence in childhood and/or adolescence show that, regardless of the type of violence and the number of occurrences, the victims suffered physical, emotional, and psychological damage. In many cases, these problems result in various clinical complications, intense suffering, and the consequent need for specialized care, which also affects their families and reaches intergenerational status^7^ ^),(^ ^11^ ^),(^ ^22^ ^),(^ ^26^ ^)-(^ ^27^. 

Thus, it can be said that sexual violence causes suffering for the victims, their families, and the healthcare team. In this respect, professional-patient interaction is necessary for care and must be mediated by empathy, an important tool for bringing victims and their families closer together^20^.

Corroborating the discussion, scholars^25^ state that it is possible to infer that the interpretation of the experiences lived by child victims can be reframed in adulthood. This possibility does not exclude the interviewees, i.e., depending on the personal life history of each professional and how they experienced (or not) situations of violence in childhood/adolescence, the meanings attributed to their role as caregiver may be compromised^12^ ^),(^ ^20^ ^),(^ ^26^ ^)-(^ ^27^, which can lead to fear.

Fear of reprisal is a common feeling among professionals who care for victims of sexual violence. This fear can be exacerbated by insecurity, physical and emotional exhaustion, a lack of adequate support for the performance of care activities and stress, which are common labor problems in national and international health services^17^ ^),(^ ^23^ ^),(^ ^28^ ^)-(^ ^29^. It should be borne in mind that sexual violence against children and adolescents can cause family members to feel so desperate and emotionally out of control that they turn against health professionals during care, as mentioned above. 

It is true that in violent contexts, society as a whole^20^, including those involved in caring for these victims, suffers shocks. For example, in Primary Health Care, professionals move around and become part of the community in response to the need to deal with the various problems through strategies such as notification in the Notifiable Diseases Information System (SINAN, in its Portuguese acronym). In this regard, a study carried out in Recife, Pernambuco, reveals that the feeling of threat can trigger fear, which is considered an impediment to compulsory notification of violence against children and young people, even though professionals recognize it as an ethical and legal obligation^30^. 

However, a national study with 242 nursing workers reveals that 20% and 59% of these professionals report having suffered physical and verbal aggression, respectively, during working hours^31^, an experience shared by nurses working in two Emergency Care Units in Paraná^32^. At the international level, a study carried out in Sweden with 1,567 health professionals indicates a prevalence of almost 25% of them experiencing violence in the workplace^33^, a reality similar to that experienced by 769 Norwegian physicians, which indicates a 20.3% prevalence of multiple threats of violence in the first four years of professional practice^29^.

The average age of 40 and the length of time the participants had been working in the health sector for more than 10 years were important elements in analyzing these results. In comparison, a study carried out in Norway states that being younger is a factor associated with professionals experiencing physical violence in their health related work^29^, a reality that differs from this study. However, the hypothesis that the professionals’ anxieties were genuine can be confirmed, given that the fear of retaliation and possible aggressive reactions from the victims’ relatives is not an unfounded feeling.

On the other hand, it is possible that this fear must be overcome during professional work to provide effective care. A study reveals that there are mechanisms for “constructing meaning” used by survivors of child sexual abuse, such as mobilizing social resources and relying on the support of society^34^. This data points to the need for health professionals to be able to act as social support for child and adolescent victims, including overcoming the aforementioned fear.

Besides, the mutual trust established between professionals is responsible for increasing the perception of safety and improving the team’s emotional control. In this way, it is possible to reduce stress and increase mutual trust in a cycle of positive reinforcement^28^. However, the lack of time and the absence of legal instruments regulating the attributions of Brazilian professionals, especially nurses, are challenges imposed on the care of victims of sexual violence^17^ and may, as a result, contribute to increasing the feeling of insecurity during healthcare.

Although it was not the result of this study, the fear of legal liability in relation to the judicial process that may be instituted after a report of sexual violence is also a feeling that affects nurses who care for victims^4^ ^),(^ ^17^. Perhaps this stems from the lack of legal instruments regulating their attributions and their role in the chain of custody of evidence^17^, which is related to the lack of a thematic approach during training and encourages future professionals to act on the front line of care for people who have suffered sexual violations^35^.

In addition to academic training, depending on the social interaction of these professionals, the symbolism attributed to sexual violence and how these professionals show their feelings, the way they react to the need to care for the victim can be nuanced. For interactionists such as Morris, social experiences influence people’s reactions and may be responsible (alongside social construction) for individuals’ actions in the face of situations they experience^20^. Therefore, the fear reported may cause professionals to feel unprepared to act in caring for victims, hindering processes such as notification and reporting, although they show indignation when they identify this type of aggression against children and adolescents. Possibly, this indignation is related to the individual’s ability as a social being to put themselves in the other person’s shoes, as Morris^20^ reveals, or simply to transfer responsibility, as denounced by professionals working at the various levels of care in Brazil^12^. In this way, indignation is a sentimental response and is related to the crime - the meaning attributed to this type of violence by health professionals, although it may not be the driving force behind care actions.

Therefore, it can be inferred that the reactions to the care given to the victims caused intense suffering among the professionals, as well as helping to make these events unforgettable, even when they occurred many years ago. In addition, emotional triggers driven by the memories experienced may be responsible for the consternation mentioned above.

In fact, assisting victims of sexual violence was pointed out as a situation that marks and shocks the entire team. Despite being difficult, professional action is based on legal prerogatives (as it is set out in the codes of ethics and the ECA) and includes notifying and reporting suspected/identified cases, actions which, if neglected, generate penalties such as fines^3^. These advances in Brazilian health care define the course of action to be taken by professionals in caring for victims, a fully systematized situation with explicit guidelines^4^, when compared to the opposite situation, such as the organization and flow of records in the Saudi Arabian health system^13^.

The development of training processes aimed at qualifying professionals to manage the process of caring for these cases is a cornerstone in the emotional preparation of professionals. Research confirms that qualifying professionals by including content in their academic training increases the possibility of them implementing pre-existing public policies^17^ ^),(^ ^35^, although this was not the case in an Asian study with 300 physicians^13^. On the other hand, the use of interactive tools promotes safe care for victims of sexual violence, reduces professionals’ feelings of helplessness and supports the clinical reasoning needed to carry out the health care process^18^. 

Given these results, it is plausible that those responsible for the occupational health program of institutions need to investigate signs and symptoms related to the mental state of professionals who work in services for victims of sexual violence, considering the negative impacts mentioned above. Furthermore, these professionals need continuous interventions in the short, medium, and long term in order to promote actions that reduce the risk of developing mild, moderate, and more serious mental disorders.

It is recommended that further studies be carried out on this subject using other methodologies. It is also suggested that this study be replicated in other contexts such as education (nurseries, schools), health services (primary care, specialized services, and maternity wards), public security services, child and adolescent protection, and justice (such as the Guardianship Council, Specialized Police Stations, the Public Prosecutor’s Office, and the Public Defender’s Office), as well as with other social groups (public security professionals, teachers, and informal caregivers) in order to compare the results obtained here and verify similarities and/or differences. 

This study is limited to revealing the feelings of female professionals who work in a hospital health service. However, these results are open to generalization, since this is a subject that causes global emotional mobilization in Western societies, which are morally organized in the light of practices that guides the protection and rights of children and adolescents. Furthermore, the study is open to replication since it used social theory and methodological rigor to ensure reliable results.

## Conclusion

Empathy, fear, indignation, suffering, and consternation are all feelings expressed by the team when caring for victims of child and adolescent sexual violence. The emphasis on these feelings makes the experience of caring for these victims a difficult and remarkable one, mobilizing feelings that can interfere with the effectiveness of the care provided.

This study reflects the reality of professionals who provide care to victims of sexual violence, so we recommend its use to qualify professionals who can deal with similar situations. Moreover, the study could guide health training on the subject and motivate the implementation of current public policies, such as the provision of psychological support for the health team responsible for caring for victims, as recommended by the Ministry of Health.
