# Supplementary material for: Feelings expressed by professionals caring for children and teenagers victims of sexual violence
Source: Rev Lat Am Enfermagem. 2024 Aug 19;32:e4251. doi: 10.1590/1518-8345.7157.4251 (PMC11335066; doi:10.1590/1518-8345.7157.4251)
Supplement: Translation

# Translation: Sentimentos expressos por profissionais no atendimento a vítimas de violência sexual infantojuvenil*

**DOI:** 10.1590/1518-8345.7157.4252

**Authors:** Marimeire Morais da Conceição, Climene Laura de Camargo, Maria Carolina Ortiz Whitaker, Camila Tahis dos Santos Silva, Nildete Pereira Gomes, Lúcia Cristina Santos Rusmando

**Affiliations:** 1 Universidade Federal da Bahia, Salvador, BA, Brasil.; 2 Secretaria de Saúde do Estado da Bahia, Escola de Saúde Pública da Bahia Professor Jorge Novis, Salvador, BA, Brasil.; 3 Bolsista da Fundação de Amparo à Pesquisa do Estado da Bahia (FAPESB), Brasil.; 4 Universidade Federal da Bahia, Escola de Enfermagem, Salvador, BA, Brasil.; 5 Bolsista do Conselho Nacional de Desenvolvimento Científico e Tecnológico (CNPq), Brasil.; 6 Universidade Federal da Bahia, Maternidade Climério de Oliveira, Salvador, BA, Brasil.

**Keywords:** Equipe de Assistência ao Paciente, Violência Doméstica, Abuso Sexual na Infância, Assistência Hospitalar, Criança, Adolescente

## Abstract

**Objetivo:**

descrever sentimentos expressos por profissionais de saúde no atendimento a vítimas infantojuvenis da violência sexual na perspectiva teórica do Interacionismo Simbólico.

**Método:**

pesquisa qualitativa realizada com 30 mulheres profissionais da saúde. Foi aplicado instrumento composto de questões fechadas para dados sociodemográficos e roteiro com perguntas abertas para a entrevista. Os dados foram organizados e analisados no *software* Nvivo versão 12, de acordo com a proposta de Bardin, sob a ótica do Interacionismo Simbólico na obra de Charles Morris. O projeto foi aprovado por Comitê de Ética em Pesquisa.

**Resultados:**

emergiram cinco categorias temáticas que revelaram sentimentos de empatia, medo, indignação, sofrimento e consternação. Tais sentimentos permaneceram na memória das entrevistadas, tornando o atendimento a vítimas infantojuvenis da violência sexual uma experiência tocante, difícil e que marca profundamente a vida do profissional de saúde.

**Conclusão:**

há necessidade de adotar estratégias de apoio à saúde mental de profissionais que atuam em serviços que prestam atendimento geral a crianças e adolescentes, considerando que existe a possibilidade de estes atenderem a vítimas infantojuvenis da violência sexual em respeito às políticas públicas preexistentes.

## Destaques

**(1)** Sentimentos oriundos de impressões individuais em consonância com a interação social.

**(2)** Atender vítimas infantojuvenis repercute negativamente nas emoções dos profissionais.

**(3)** Gestores do serviço precisam atentar para a saúde de profissionais que atendem às vítimas.

## Introdução

A violência sexual infantojuvenil (VSI) engloba uma multiplicidade de condutas de natureza sexual com crianças e adolescentes, sejam elas consensuais ou não^1^ ^)-(^ ^2^. No Brasil, este conceito foi classificado em três categorias: abuso sexual; exploração sexual comercial e o tráfico de pessoas^3^ ^)-(^ ^4^, que inclui modalidades propagadas com o uso da Internet, como *sexting*, sextorsão e estupro virtual^5^, o que corrobora posicionamentos internacionais acerca da temática^1^ ^),(^ ^5^.

Segundo a Organização das Nações Unidas (ONU)^6^ ^)^ e a Organização Mundial da Saúde (OMS)^5^, um bilhão de crianças e adolescentes no mundo com idade entre dois e 17 anos são vitimizadas. Estudo revela a prevalência de violência sexual de 14,5% a 89,4% entre 227 adolescentes africanos^7^; similarmente, no Brasil, o Boletim Epidemiológico recente alerta que a violência sexual é mais perpetrada contra crianças com idade entre cinco e nove anos e adolescentes entre 12 e 14 anos do sexo feminino^8^. 

Em que pesem estes dados alarmantes, a invisibilidade dos casos é uma constatação. Estudos internacionais apontam para a dificuldade de identificação dos casos^9^ ^)-(^ ^12^, consequentemente, infere-se que o cuidado profissional às vítimas, ainda hoje, consiste em um desafio potencializado por tabus impostos socialmente em diversos contextos culturais. Esta situação é confirmada em estudo realizado na Arábia Saudita com 300 médicos da Atenção Primária à Saúde que, embora tenham conhecimento sobre abusos infantis, não registram os casos, o que gera subnotificação nos sistemas do país^13^. 

Estudos salientam que a assistência às vítimas envolve humanização no acolhimento e prevenção de outros agravos; bem como o acompanhamento psicossocial e de saúde^14^ ^)-(^ ^17^, processo aprimorado recentemente no país através do Estatuto da Criança e do Adolescente (ECA)^3^ ^)-(^ ^4^. Soma-se a este enfrentamento a disponibilidade de dispositivos tecnológicos para a denúncia, porém sua existência não dispensa o estabelecimento de interações humanas^4^ ^),(^ ^18^ ^)-(^ ^19^. 

Sabe-se que, para interagir, o indivíduo necessita ter contato (visual, auditivo, táctil e/ou verbal) com o outro, a fim de identificar situações, pessoas e objetos que são alvo de esforços, ações e intervenções^20^. Portanto, o atendimento às vítimas e suas famílias é mediado pelos sentimentos, considerados molas propulsoras das ações sempre que há atribuição de significados a esta interação^19^ ^)-(^ ^20^, cujas influências são marcadas pela convivência em sociedade^20^ ^)-(^ ^21^. 

O atendimento às vítimas infantojuvenis da violência sexual requer preparo emocional, pois o lidar com os sentimentos durante este atendimento consiste em outro desafio. Estudo revela um cenário impactante percebido por profissionais ao assistir crianças e adolescentes vitimizados que apresentam lacerações de órgãos genitais, sangramentos intensos, condições de saúde que exprimem grave complicação clínica e podem culminar em óbito^22^. Estas situações são marcantes para a experiência humana e podem ocasionar dificuldades na atuação da equipe de saúde. 

Isto se explica porque as intervenções necessárias no atendimento às vítimas precisam ser mediadas por interações empáticas que mexem com estes sentimentos^19^. Por outro lado, a literatura revela que profissionais de um centro de atenção psicossocial expressam sentimentos como impotência e frustração ao cuidarem de crianças vítimas de violência^23^, porém permanece a lacuna no conhecimento em relação aos sentimentos expressos por profissionais presentes no atendimento a vítimas de violência sexual infantojuvenil.

A relevância deste estudo está na possibilidade de contribuir para a melhoria do preparo emocional de profissionais que atuam em serviços da saúde em qualquer nível de atenção. Esta medida colabora para implementar recomendações do Ministério da Saúde para que estes trabalhadores sejam preparados e envolvidos em prol dos cuidados às vítimas e suas famílias^4^. No entanto, também serve para subsidiar políticas de saúde ocupacional nos serviços de saúde e, consequentemente, ampliar o alcance destas políticas para os diversos contextos de atendimento a crianças e adolescentes, como escolas, creches, Conselhos Tutelares e Delegacias Especializadas, entre outros, visto que os sentimentos evidenciados podem ser abrangentes a pessoas que interagem e estão em convívio na mesma sociedade. Logo, este estudo tem como objetivo descrever sentimentos expressos por profissionais de saúde no atendimento a vítimas infantojuvenis de violência sexual na perspectiva teórica do Interacionismo Simbólico.

## Método

### Tipo de estudo

Estudo qualitativo apresentado segundo a ferramenta *Consolidated Criteria for Reporting Qualitative Research* (COREQ) adaptada para o Brasil^24^, produto de uma dissertação de mestrado defendida em 2020 em um programa de pós-graduação do país. 

### Local do estudo

A pesquisa ocorreu em um hospital público do Nordeste brasileiro que fornece atendimento geral, ambulatorial, clínico, cirúrgico, de urgência/emergência, para todos os grupos etários e de ambos os sexos. A aproximação com o campo ocorreu por meio da atuação da primeira autora como enfermeira no referido hospital. Foram visitadas as unidades assistenciais abertas (clínicas) e fechadas (unidades de atendimento a pacientes críticos), voltadas ao público pediátrico e adolescente. O projeto foi apresentado à diretoria, a responsáveis por unidades assistenciais e a possíveis participantes em um encontro prévio.

### Participantes do estudo

Para a seleção dos participantes, todos os profissionais de saúde encontrados nas unidades assistenciais foram abordados individualmente e convidados a integrar a pesquisa. Mediante o aceite, eram conferidos os critérios de inclusão: atuar por mais de um ano na instituição e ter prestado assistência a crianças e adolescentes que sofreram violência sexual; e os critérios de exclusão: estar ausente do serviço pesquisado por quaisquer motivos e laborar em unidades de apoio diagnóstico. 

Não houve recusas/desistências dos convidados em integrar a pesquisa. Desta maneira, dos 36 profissionais abordados aleatoriamente, apenas 31 respondiam aos critérios de inclusão, porém uma entrevistada foi descartada devido à insuficiência de informação.

### Coleta de dados

A coleta de dados ocorreu em junho e julho de 2019, conduzida pela mestranda em companhia de quatro acadêmicas de Enfermagem (todas do sexo feminino) que participavam individualmente da entrevista na função de conferir o cumprimento do roteiro previsto. Todas as estudantes eram vinculadas a um grupo de pesquisa, devidamente qualificadas para coleta de dados qualitativos em um curso de extensão frequentado no mesmo ano. 

Inicialmente, aplicou-se um questionário estruturado e um roteiro de entrevista semiestruturada previamente criado pela pesquisadora que incluía a seguinte questão: como você se sentiu durante o atendimento às vítimas? Porém, ao longo dos relatos, as profissionais mencionavam também as reações observadas em outros profissionais percebidas ao longo do processo assistencial às vítimas. 

As entrevistas em profundidade foram pré-agendadas com as participantes. Os diálogos, que ocorreram uma única vez, em horário de trabalho das profissionais (períodos diurno e noturno) e tiveram duração média de 45 min, foram audiogravados. Cabe destacar que a saturação temática dos dados foi identificada na 23ª participante e confirmada com mais sete entrevistas que apresentavam similaridade das experiências compartilhadas, respondiam ao objetivo proposto e não apresentavam acréscimos para a interpretação e análise dos dados.

### Análise de dados

Após a coleta, foi realizada a transcrição pelas estudantes, porém, apenas uma participante concordou em ler e corrigir sua entrevista transcrita. Diante desta dificuldade, a correção e a validação textual das demais participantes foram realizadas pela pesquisadora responsável. Utilizou-se a técnica de análise de conteúdo temática sugerida por Bardin^25^ em três etapas: a) pré-análise; b) exploração do material; c) tratamento dos resultados e inferência.

Na etapa de pré-análise, as entrevistas foram transcritas, impressas e lidas superficialmente na íntegra. A seguir, procedeu-se à extração de temas preliminares que suscitaram a menção a sentimentos expressos que estavam também presentes nas observações das pesquisadoras, nas discussões após cada entrevista e que foram apontadas nos registros do diário reflexivo. 

A segunda fase - exploração do material - foi iniciada manualmente. Foi realizado um grupo de reflexão que, semanalmente, se reunia para analisar, discutir, refletir sobre e compartilhar as impressões e interpretações dos dados. Os textos transcritos foram submetidos a releituras persistentes (cada entrevista foi lida, no mínimo, oito vezes pela pesquisadora principal e três vezes pelas acadêmicas). Neste processo, foram realizadas leituras aprofundadas, fase que ocorreu concomitantemente à escuta reiterada (mínimo de cinco vezes) de cada entrevista, a fim de possibilitar a impregnação dos textos pelas pesquisadoras. Após este processo, os textos foram importados para o *software* NVivo versão 12, onde foram organizados e sistematizados em códigos, ou nós temáticos.

Para proceder à terceira etapa - interpretação - as falas das participantes foram agrupadas em códigos por informações convergentes, dando origem ao total de 23 expressões e termos que foram considerados subtemas ou subcategorias, agrupados por significado convergente em categorias temáticas^25^. A validação do processo de análise se deu a partir de discussões com três acadêmicas de Enfermagem e três pesquisadoras doutoras, além de um momento reservado com cinco participantes da pesquisa que confirmaram os resultados apresentados. O processo de análise dos dados é descrito na Figura 1. 

**Figura 1:** Exemplos de expressões que originaram as categorias temáticas na perspectiva do Interacionismo Simbólico. Salvador, BA, Brasil, 2019

Objeto simbólico	Expressões evidenciadas nas interações sociais	Significado	Significante
Criança/adolescente violentada sexualmente	“*poderia ser seus filhos*”	Ponderação acerca do seu possível sofrimento ao vivenciar uma situação semelhante (ter o filho criança/adolescente violentado) e sopesar o sofrimento que o outro pode estar vivenciando ao ter a filha violentada	Empatia
	“*pensa o sofrimento da mulher com a filha nesse estado*”		
	“*me coloco no lugar de mulher*”		
Ação de buscar informações para elucidar suspeita de violência sexual	“*receosas de fazer muita pergunta e mãe reclamar*”	Possíveis reações que familiares das vítimas teriam frente às perguntas relacionadas à suspeita de violência sexual	Medo
	“*medo de dizer suspeita ... família ameaça*”		
Ações do agressor e reações frente à violência sexual contra crianças/adolescentes	*“indignado”*	Reação perante agressões sexuais infantojuvenis é rechaçar o agressor. Converge a opinião quanto à necessidade de insurgência e intolerância da sociedade contra este ato e contra aqueles que não expressam o mesmo sentimento	Indignação
	“*me ressentia ... mãe não demonstrou raiva*”		
Apresentação de quadro clínico complexo que demanda esforços físicos e emocionais dos profissionais	“*médico chorava muito* [...] *me senti péssima*”	Reação em reflexo à percepção de que a vítima também sofre ou mesmo resultado dos esforços físicos e emocionais empreendidos ao longo do atendimento	Sofrimento
	“*como se minha energia tivesse sido sugada*”		
Longo período após o momento do atendimento	“*até hoje, fica na minha mente* [...] *todo mundo fica emocionalmente triste*”	Reação de desalento aprofundado diante de alguma situação mobilizadora; esta reação pode perdurar independente da perda (e/ou distanciamento *físic*o) ou mesmo do afastamento emocional de um objeto social, nestes casos, a história da vítima	Consternação
	“*levo um tempo processando essas histórias* [...] *não tem como não ficar mobilizada, triste*”		

As análises, inferências e interpretações recomendadas por Bardin^25^ estão ancoradas nas bases filosóficas do Interacionismo Simbólico (IS), presentes na obra “Mente, *self* e sociedade” de Charles Morris. Esta teoria tem como precursor o cientista social George Herbert Mead, que teve suas ideias sistematizadas por seu orientando de doutorado Herbert Blumer. Durante o século XIX, o IS foi incorporado à Psicologia Social, conduzindo o arcabouço teórico constituído por três premissas basais: 1) a forma como uma pessoa interpreta os fatos e age depende do significado que este lhe é atribuído; 2) o significado é edificado a partir de processos interativos em sociedade; e 3) ao longo do tempo, os significados podem sofrer alterações^20^.

Esta teoria centraliza o olhar nas interações sociais, nas respostas humanas e na influência que a imersão do indivíduo na sociedade tem nos seus posicionamentos frente às situações experienciadas^20^. Assim, foram evidenciadas cinco categorias temáticas: empatia, medo, indignação, sofrimento e consternação.

### Aspectos éticos

O projeto foi aprovado pelo Comitê de Ética em Pesquisa e respeita as Resoluções do Conselho Nacional de Saúde (466/2012 e 510/2016). Desta maneira, todas as pessoas que integraram a pesquisa, após leitura e concordância, assinaram as duas vias do Termo de Consentimento Livre e Esclarecido. 

As entrevistas foram feitas em salas privativas, localizadas na instituição pesquisada, em presença da pesquisadora principal e uma acadêmica de Enfermagem, com o intuito de garantir confidencialidade e sigilo às informações prestadas. Para preservar o anonimato das participantes, seus nomes foram substituídos por “P” (referente a profissional), seguido de um número arábico, conforme ordem de realização das entrevistas: P1, P2, P3 ... P30.

## Resultados

Participaram 30 profissionais de saúde, sendo 33,3% enfermeiras, 33,3% técnicas de enfermagem, 10,0% assistentes sociais, 10,0% médicas, 6,7% auxiliares de enfermagem e 6,7% psicólogas. A totalidade das participantes referiu ser do sexo feminino, autorreferidas de identidade de gênero mulher cis, orientação afetivo-sexual heterossexual, 80% da raça/cor negra e tinham idade entre 25 e 65 anos. Ainda, 73,3% tinham mais de dez anos de formação profissional concluída, 70% atuavam há mais de dez anos na área da saúde e 40% tinham mais de dez anos de atuação no hospital estudado. 

Após o processo analítico, emergiram cinco categorias temáticas que representam os sentimentos expressos por profissionais e evidenciados nos relatos, a saber: empatia, medo, indignação, sofrimento e consternação, como descrito a seguir.

### Empatia

De acordo com os relatos das participantes, o sentimento mais evocado foi a empatia. Empatia significa ter a capacidade de colocar-se no lugar do outro indivíduo sem, necessariamente, passar pelas mesmas experiências que ele. A empatia é considerada um sentimento relevante e indispensável ao cuidado, como foi mencionado:

*A agressão sexual contra uma criança ou adolescente interfere muito no emocional. Você logo pensa que poderia ser seus filhos* (P11, Técnica de enfermagem)*.*

*O coração acelera! A gente fica vendo a situação do ser humano, dessa criança/adolescente. Não sei como alguém é capaz de pegar uma criança, um bebê, como já aconteceu. Eu me pergunto: o que está acontecendo com o ser humano?* [...] (P26, Assistente social).

*Maciçamente a mulher é vítima de estupro. Isso mexe com nosso lugar de mulher, que é subjugada. A gente pensa no sofrimento dessa mulher, mãe, avó ao ver a filha nesse estado* (P12, Assistente Social)

*Eu me coloco muito no lugar de mulher. Para mim, seria a pior coisa que eu poderia sofrer na minha vida.* [...] (P13, Enfermeira).

### Medo

O medo foi outro sentimento expresso pelos profissionais em estudo que ocorria, principalmente, durante a coleta de informações sobre o caso de violência sexual. Esta coleta de informações é feita nos serviços de saúde com base nos relatos dos responsáveis pelas vítimas, na realização de exames laboratoriais, de imagem e anamnese. O medo também ocorria expressamente durante o ato de comunicar o diagnóstico (ou mesmo a suspeita dele) aos familiares das vítimas:

[...] *o local sangrava e estava muito aberto. Ficamos receosas de fazer muita pergunta e a mãe reclamar na diretoria do hospital dizendo que estávamos perguntando demais, constrangendo-a!* (P1, Técnica de enfermagem)*,*

*Ficamos com medo de dizer que existe suspeita de violência sexual porque, muitas vezes, a família ameaça a equipe* (P19, Técnica de enfermagem).

*Quando eu e a outra técnica fomos trocar a fralda dele e fazer higiene íntima, observamos que o ânus estava muito dilatado.* [...] *tivemos receio de dizer qualquer coisa acerca da suspeita e o pai reagir mal* (P10, Técnica de enfermagem).

### Indignação

As colaboradoras relatam indignação, revolta e desespero diante do atendimento às vítimas de violência sexual, seja ela criança ou adolescente. O sentimento é tão aflorado que, em alguns casos, rebelam-se contra o agressor:

[...] *um menino de um ano e seis meses choroso, assustado. A médica constatou fissuras, queloides e sangramento no reto. Eu e a anestesista ficamos muito indignadas!* (P2, Auxiliar de enfermagem).

[...] *o parente mostrava a genitália, ameaçava ela [a adolescente] de morte caso ela contasse aos pais. Um absurdo! Eu estava torcendo para o agressor morrer!* (P14, Enfermeira).

[...] *era uma menina inocente, que nem sabia o que tinha acontecido com ela. Lacerou a genitália. O que mais me ressentia era por causa da mãe que, em momento nenhum, demonstrou raiva* (P9, Técnica de enfermagem).

*O* [médico] *anestesista ficou indignado! A equipe toda estava indignada!* (P8, Técnica de enfermagem)*.*

### Sofrimento

As profissionais entrevistadas relatam que se sentiram tão impactadas diante dos casos de violência sexual infantojuvenil, a ponto de diversos membros da equipe apresentarem o choro como a principal expressão da emoção vivenciada (misto de revolta, tristeza, raiva, empatia com a vítima). O sofrimento das vítimas refletia e gerava sofrimento também nos profissionais durante o atendimento:

*A criança tinha uns dois anos.* [...] *lesionou da genitália até o ânus. O médico chorava muito enquanto fazia a cirurgia. Eu me senti péssima!* (P9, Técnica de enfermagem)*.*

*Eu e o cirurgião ficamos muito chocados, pois era uma criança de oito meses que chegou em estado muito grave, com lesões importantes no ânus e vagina. Fiquei muito abalada emocionalmente!* (P25, Médica)*.*

[...] *são casos difíceis que envolvem o nosso sentimento. Depois do atendimento, me sinto esgotada, como se minha energia tivesse sido sugada* (P27, Psicóloga)*.*

### Consternação

De forma recorrente, a imagem das vítimas e lembranças das suas histórias estão presentes nas memórias das profissionais, despertando o sentimento de consternação. Segundo as participantes, isso ocorria mesmo após ter passado um longo período em que prestaram assistência a estas crianças e/ou adolescentes:

*Aconteceu há uns dois anos atrás e, até hoje, fica na minha mente quando essa criança chegou com a região perianal sangrando e foi para a sala de cirurgia* [...] *até hoje, todo mundo fica emocionalmente triste* (P1, Técnica de enfermagem)*.*

*Cuidei de uma criança, que tinha menos de um mês de vida e foi a óbito.* [...] *em casa, eu só conseguia pensar nisso. Durante muito tempo, sempre que estava trabalhando, eu olhava para o leito e só pensava nela* [...] *e fica todo mundo muito triste com essa lembrança* (P23, Auxiliar de enfermagem)*.*

*A mãe trouxe a menina para a emergência, ela suspeitava que o agressor era uma pessoa da família.* [...] *quando atendo casos de abuso sexual em crianças e adolescentes, levo um tempo processando essas histórias porque não tem como você não ficar muito mobilizada, triste, após atender um caso desse* (P12, Assistente social)*.*

## Discussão

De acordo com os relatos das participantes, os sentimentos mais evocados foram: a empatia pelas vítimas; o medo de informar a suspeita de violência sexual, de direcionar o atendimento para este agravo e de ser retaliada; a indignação com relação ao fato ocorrido (violência sexual contra crianças e adolescentes) e com a inércia inesperada da família da vítima; o sofrimento diante dos casos clínicos e das situações de cuidado experienciadas; a consternação em relação *à vítima, aos fatos relatados e* ao cuidado prestado.

Estes achados alertam para a complexidade que se instaura no indivíduo envolvido no atendimento a crianças e adolescentes vitimizadas sexualmente. Embora a VSI esteja posta na sociedade e isto seja evidente em diversos estudos^7^ ^)-(^ ^12^, sua constatação na vida real ainda mobiliza as emoções de profissionais atuantes a ponto destes resgatarem em si sentimentos que podem lev*á-los* à completa paraliza*ção* ou dar início a ações de cuidado necessárias, processo que depende da percepção de mundo de cada um. 

Segundo Morris, a percepção é uma espécie de mediador necessário às interações entre as pessoas^20^. Nesta perspectiva, para a prestação de cuidados, é preciso ter empatia, o que possibilita criação de laços de afeto, compreensão e reciprocidade afetiva. Isso porque, ao utilizar da empatia para prestar cuidados às vítimas infantojuvenis de violência sexual, os profissionais de saúde envolvem os sentimentos de fraternidade e compaixão, considerados importantes para o cuidado humano^21^. A empatia é um sentimento que atribui ao indivíduo a capacidade psicológica de sentir o que sentiria a outra pessoa, caso estivesse na mesma situação vivenciada por ela^19^ ^)-(^ ^21^, o que ocorre por meio de interações. 

Estudos nacionais e internacionais sobre a vivência de violência doméstica na infância e/ou adolescência apontam que, independentemente do tipo de violência e do número de ocorrências, as vítimas tiveram prejuízos físicos, emocionais e psíquicos. Em muitos casos, estes agravos resultam em complicações clínicas diversas, sofrimento intenso e consequente necessidade de atendimento especializado, que abarcam também seus familiares e alcançam status intergeracional^7^ ^),(^ ^11^ ^),(^ ^22^ ^),(^ ^26^ ^)-(^ ^27^. 

Assim, pode-se afirmar que a violência sexual ocasiona sofrimento às vítimas, seus familiares e à equipe de saúde. Neste tocante, a interação profissional-paciente é necessária à assistência e deve ser mediada pela empatia, importante instrumento de aproximação e acolhimento às vítimas e seus familiares^20^.

Corroborando a discussão, estudiosos^25^ afirmam que é possível inferir que a interpretação das experiências vivenciadas pelas vítimas infantis possam ser ressignificadas na idade adulta. Esta possibilidade não exclui as entrevistadas, ou seja, a depender do histórico de vida pessoal de cada profissional e de como cada uma vivenciou (ou não) situações de violência na infância/adolescência, os significados atribuídos ao seu papel de cuidador podem estar comprometidos^12^ ^),(^ ^20^ ^),(^ ^26^ ^)-(^ ^27^, o que pode ocasionar medo.

O medo de represália é sentimento comum entre profissionais que cuidam de vítimas da violência sexual. Este medo pode ser incrementado pela insegurança, esgotamento físico e emocional, falta de suporte adequado ao desempenho de atividades de cuidado e estresse, problemas laborais frequentes nos serviços de saúde nacionais e internacionais^17^ ^),(^ ^23^ ^),(^ ^28^ ^)-(^ ^29^. Há de se ponderar que a violência sexual infantojuvenil pode despertar nos familiares sentimento de desespero e descontrole emocional a ponto de se insurgirem contra os profissionais durante o atendimento, como foi mencionado. 

É certo que, em contextos violentos, toda a sociedade^20^, incluindo aqueles envolvidos nos cuidados a estas vítimas, sofre abalos. Como exemplo, na Atenção Primária à Saúde, profissionais circulam e se incorporam à comunidade em resposta à necessidade de enfrentar os diversos agravos por meio de estratégias como a notificação no Sistema de Informação de Agravos de Notificação (Sinan). Sobre isto, estudo realizado em Recife, Pernambuco, revela que a sensação de ameaça pode desencadear o sentimento de medo, considerado impeditivo para a realização da notificação compulsória de violência infantojuvenil, embora os profissionais reconheçam sua efetivação como obrigação ética e legal^30^. 

Contudo, um estudo nacional com 242 trabalhadores de enfermagem revela que 20% e 59% destes profissionais denunciam ter sofrido, respectivamente, agressões físicas e verbais nos horários de labor^31^, experiência compartilhada por enfermeiros do quadro funcional de duas Unidades de Pronto Atendimento (UPA) no Paraná^32^. No âmbito internacional, estudo realizado na Suécia com 1.567 profissionais da saúde aponta prevalência de quase 25% de vivência de violência no local de trabalho^33^, realidade similar àquela vivida por 769 médicos noruegueses, que aponta 20,3% de prevalência de múltiplas ameaças de violência nos quatro primeiros anos de atuação profissional^29^.

A idade média de 40 anos e o tempo de atuação profissional superior a 10 anos na área da saúde mencionados pelas participantes foram elementos importantes para a análise destes resultados. Em comparação, estudo desenvolvido na Noruega afirma que ter menor idade é fator associado à vivência de violência física por profissionais na prática laboral em saúde^29^, realidade que diverge desta pesquisa. No entanto, pode-se confirmar a hipótese de que os anseios das profissionais eram genuínos, visto que o medo de sofrer retaliações e de possíveis reações agressivas de parentes das vítimas não é um sentimento infundado.

Por outro lado, é possível que este medo tenha que ser superado durante a atuação profissional em prol da efetividade da assistência. Estudo revela que existem mecanismos de “construção de significado” usados por sobreviventes de abusos sexuais infantis, tais como mobilizar recursos sociais e contar com o alicerce da sociedade^34^. Estes dados alertam para a necessidade de profissionais da saúde estarem aptos a atuar como suporte social para as vítimas infantojuvenis, inclusive superando o medo mencionado.

Para além destes, a confiança mútua estabelecida entre os profissionais é a responsável por ampliar a percepção de segurança e melhorar o controle emocional da equipe. Desta maneira, é possível diminuir o estresse e aumentar a confiança mútua em um ciclo de reforço positivo^28^. Entretanto, a falta de tempo e a ausência de instrumentos legais que regulamentem as atribuições dos profissionais brasileiros, sobretudo a enfermagem, são desafios impostos nos cuidados às vítimas de violência sexual^17^, podendo, em consequência, contribuir para ampliar o sentimento de insegurança durante o cuidado.

Embora não tenha sido resultado deste estudo, o medo da responsabilidade legal com relação ao processo judicial que se pode instaurar após a denúncia de violência sexual também é um sentimento que acomete enfermeiros que cuidam das vítimas^4^ ^),(^ ^17^. Talvez isto decorra da ausência de instrumentos legais que regulamentem suas atribuições e seu papel na cadeia de custódia das provas^17^, o que está relacionado à falta de abordagem temática durante a formação e estimula futuros profissionais a atuarem na linha de frente de cuidados a pessoas que sofreram violações sexuais^35^.

Para além da formação acadêmica, a depender do convívio social destes profissionais, da simbologia atribuída à violência sexual e de como esses profissionais demonstram seus sentimentos, a forma de reagir frente à necessidade de cuidar da vítima pode apresentar nuances. Para os interacionistas como Morris, as experiências sociais influenciam as reações das pessoas, podendo ser as responsáveis (ao lado da construção social) pelas ações dos indivíduos frente às situações vivenciadas^20^. Portanto, o medo relatado pode ocasionar o sentimento de despreparo do profissional para atuar no cuidado à vítima, dificultar processos como a notificação e a denúncia, embora eles demonstrem indignação quando identificam este tipo de agressão contra crianças e adolescentes. Possivelmente, esta indignação está relacionada à capacidade que o indivíduo como ser social tem de se colocar no lugar do outro, como revela Morris^20^, ou simplesmente transferir a responsabilidade, como denunciam profissionais atuantes nos diversos níveis de assistência no Brasil^12^. Desta maneira, a indignação é resposta sentimental e está relacionada ao crime - significado atribuído a este tipo de violência para profissionais da saúde, embora possa não ser elemento impulsionador de ações de cuidado. 

Portanto, infere-se que as reações diante do atendimento às vítimas ocasionaram sofrimento intenso e aflorado entre os profissionais, bem como contribuíram para tornar os fatos vivenciados inesquecíveis, mesmo quando ocorreram há muitos anos. Ademais, gatilhos emocionais impulsionados a partir das memórias vividas podem ser os responsáveis pela consternação mencionada.

Aliás, o atendimento a vítimas de violência sexual foi apontado como uma situação que marca e choca toda a equipe. Apesar de difícil, a atuação profissional está embasada em prerrogativas legais (pois consta nos códigos de deontologia, no ECA), abarca a notificação e a denúncia dos casos suspeitos/identificados, ações que, se negligenciadas, geram penalidades, como multas^3^. Estes avanços na saúde brasileira definem a condução a ser tomada por profissionais no atendimento às vítimas, situação plenamente sistematizada com orientações explícitas^4^, quando se compara com situação oposta, como a organização e fluxo de registros do sistema de saúde da Arábia Saudita^13^.

O desenvolvimento de processos formativos que visem qualificações para a condução do processo de cuidar destes casos é alicerce no preparo emocional de profissionais. Pesquisas confirmam que qualificar profissionais através da inserção de conteúdos na formação acadêmica ampliam a possibilidade de estes implementarem políticas públicas pré-existentes^17^ ^),(^ ^35^, ainda que esta não seja uma constatação em um estudo asiático com 300 médicos^13^. Por outro lado, o uso de ferramenta interativa promove a assistência segura às vítimas de violência sexual, reduz o sentimento de impotência do profissional e dá suporte ao raciocínio clínico necessário à execução do processo assistencial de saúde^18^. 

Diante destes resultados, é plausível que os responsáveis pelo programa de saúde ocupacional das instituições precisem investigar sinais e sintomas relativos ao estado mental de profissionais que atuam em serviços de atendimento a vítimas de violência sexual, considerando os impactos negativos acima mencionados. Ainda, estes profissionais necessitam de intervenções contínuas, a curto, médio e longo prazo, a fim de promover ações que reduzam os riscos de desenvolver transtornos mentais leves, moderados e com repercussões de maior gravidade.

Recomenda-se a realização de outros estudos sobre esta temática, que utilizem outras metodologias. Também sugere-se que o presente estudo seja replicado em outros contextos como da educação (creches, escolas), em serviços de saúde (da atenção primária, serviços especializados e maternidades), serviços da segurança pública, e na proteção à infância e adolescência, e justiça (como Conselho Tutelar, Delegacias Especializadas, Ministério Público, e Defensoria Pública), bem como com outros grupos sociais (profissionais da segurança pública, professores e cuidadores informais) para fins de confrontar os resultados aqui obtidos e verificar semelhanças e/ou diferenças. 

Este estudo limita-se a desvelar sentimentos de mulheres profissionais que atuam em um serviço hospitalar de saúde. Entretanto, estes resultados são passíveis de generalização, posto que se trata de uma temática que ocasiona mobilização emocional de forma global em sociedades ocidentais, organizadas moralmente à luz de condutas que orientam a proteção e o direito de crianças e adolescentes. Ademais, o estudo é passível de replicação, visto que se recorreu a uma teoria social e rigor metodológico para assegurar resultados fidedignos.

## Conclusão

Empatia, medo, indignação, sofrimento e consternação são sentimentos expressos pela equipe no atendimento às vítimas de violência sexual infantojuvenil. A ênfase destes sentimentos torna a experiência de cuidar destas vítimas uma vivência difícil, marcante e que mobiliza sentimentos que podem interferir na efetividade dos cuidados prestados.

Este estudo reflete a realidade de profissionais que prestam assistência às vítimas de violência sexual, portanto, recomendamos seu uso para qualificar profissionais que possam lidar com situações semelhantes. Ademais, o estudo poderá nortear a formação em saúde acerca da temática, motivar a efetivação de políticas públicas vigentes, tal qual a oferta de acompanhamento psicológico para a equipe de saúde responsável pelo cuidado às vítimas, como preconiza o Ministério da Saúde.

# Translation: Sentimientos expresados por los profesionales que atienden a víctimas de violencia sexual infantojuvenil*

**DOI:** 10.1590/1518-8345.7157.4250

**Authors:** Marimeire Morais da Conceição, Climene Laura de Camargo, Maria Carolina Ortiz Whitaker, Camila Tahis dos Santos Silva, Nildete Pereira Gomes, Lúcia Cristina Santos Rusmando

**Affiliations:** 1 Universidade Federal da Bahia, Salvador, BA, Brasil.; 2 Secretaria de Saúde do Estado da Bahia, Escola de Saúde Pública da Bahia Professor Jorge Novis, Salvador, BA, Brasil.; 3 Becaria de la Fundação de Amparo à Pesquisa do Estado da Bahia (FAPESB), Brasil.; 4 Universidade Federal da Bahia, Escola de Enfermagem, Salvador, BA, Brasil.; 5 Becaria del Conselho Nacional de Desenvolvimento Científico e Tecnológico (CNPq), Brasil.; 6 Universidade Federal da Bahia, Maternidade Climério de Oliveira, Salvador, BA, Brasil.

**Keywords:** Grupo de Atención al Paciente, Violencia Doméstica, Abuso Sexual Infantil, Atención Hospitalaria, Niño, Adolescente

## Abstract

**Objetivo:**

describir los sentimientos expresados por los profesionales de la salud en la atención a víctimas infantojuveniles de violencia sexual desde la perspectiva teórica del Interaccionismo Simbólico.

**Métodos:**

investigación cualitativa realizada con 30 profesionales de salud. Se utilizó un instrumento compuesto por preguntas cerradas para los datos sociodemográficos y un guión con preguntas abiertas para la entrevista. Los datos fueron organizados y analizados utilizando el *software* Nvivo versión 12, de acuerdo con la propuesta de Bardin, desde la perspectiva del Interaccionismo Simbólico en la obra de Charles Morris. El proyecto fue aprobado por el Comité de Ética de la Investigación.

**Resultados:**

surgieron cinco categorías temáticas que revelaron sentimientos de empatía, miedo, indignación, sufrimiento y consternación. Estos sentimientos permanecieron en la memoria de los entrevistados, convirtiendo la atención a infantojuveniles víctimas de violencia sexual en una experiencia conmovedora y difícil que marca profundamente la vida del profesional de salud.

**Conclusión:**

es necesario adoptar estrategias de apoyo a la salud mental de los profesionales que actúan en servicios de atención general a infantojuveniles, considerando que existe la posibilidad de que atiendan a infantojuveniles víctimas de violencia sexual en cumplimiento de políticas públicas preexistentes.

## Destacados

**(1)** Los sentimientos surgen de las impresiones individuales en función de la interacción social.

**(2)** La atención a víctimas infantojuveniles tiene un impacto negativo en las emociones de los profesionales.

**(3)** Los gestores de los servicios deben prestar atención a la salud de los profesionales que atienden a las víctimas.

## Introducción

La violencia sexual infantojuvenil (VSI) engloba una multiplicidad de comportamientos sexuales hacia infantojuveniles, consentidos o no^1^ ^)-(^ ^2^. En Brasil, este concepto ha sido clasificado en tres categorías: abuso sexual; explotación sexual comercial y tráfico de personas^3^ ^)-(^ ^4^, que incluye modalidades propagadas a través del uso de Internet, como el sexting, la sextorsión y la violación virtual^5^, lo que corrobora las posiciones internacionales sobre el tema^1^ ^),(^ ^5^. 

Según la Organización de las Naciones Unidas (ONU)^6^ y la Organización Mundial de la Salud (OMS)^5^, mil millones de infantojuveniles de entre 2 y 17 años son víctimas de violencia en el mundo. Un estudio revela una prevalencia de la violencia sexual de entre el 14,5% y el 89,4% entre 227 adolescentes africanos^7^; asimismo, en Brasil, el reciente Boletín Epidemiológico advierte de que la violencia sexual se ejerce con mayor frecuencia contra los niños de entre cinco y nueve años y adolescentes de entre 12 y 14 años del sexo femenino^8^. 

A pesar de estas cifras alarmantes, la invisibilidad de los casos es un hecho. Estudios internacionales señalan la dificultad para identificar los casos^9^ ^)-(^ ^12^, lo que significa que la atención profesional a las víctimas sigue siendo un reto, alimentado por tabúes socialmente impuestos en diversos contextos culturales. Esta situación se confirma en un estudio realizado en Arabia Saudí con 300 médicos de atención primaria que, aunque conocen los casos de maltrato infantil, no los registran, lo que provoca un subregistro en los sistemas del país^13^. 

Estudios señalan que la asistencia a las víctimas pasa por la humanización de la acogida y la prevención de otros problemas, así como por el acompañamiento psicosocial y de salud^14^ ^)-(^ ^17^, proceso que ha sido mejorado recientemente en el país a través del Estatuto del Niño y del Adolescente (ECA, por su acrónimo en portugués)^3^ ^)-(^ ^4^. A esto se suma la disponibilidad de dispositivos tecnológicos para la denuncia, pero su existencia no prescinde del establecimiento de interacciones humanas^4^ ^),(^ ^18^ ^)-(^ ^19^. 

Se sabe que para interactuar, los individuos necesitan tener contacto (visual, auditivo, táctil y/o verbal) con otros para identificar situaciones, personas y objetos que son blanco de esfuerzos, acciones e intervenciones^20^. Por lo tanto, la atención a las víctimas y sus familiares está mediada por los sentimientos, que se consideran el motor de las acciones siempre que haya una atribución de significado a esta interacción^19^ ^)-(^ ^20^, cuyas influencias están marcadas por la convivencia en sociedad^20^ ^)-(^ ^21^.

Atender a infantojuveniles víctimas de violencia sexual requiere preparación emocional, pues lidiar con los sentimientos durante esa atención es otro desafío. Un estudio revela un escenario impactante percibido por los profesionales al asistir a infantojuveniles victimizados que presentan laceraciones genitales, hemorragias intensas, cuadros de salud que expresan complicaciones clínicas graves y pueden culminar en muerte^22^. Estas situaciones están marcadas por la experiencia humana y pueden causar dificultades en el trabajo del equipo de salud. 

Esto puede explicarse por el hecho de que las intervenciones necesarias para atender a las víctimas deben estar mediadas por interacciones empáticas que toquen estos sentimientos^19^. Por otro lado, la literatura muestra que los profesionales de un centro de atención psicosocial expresan sentimientos como impotencia y frustración cuando atienden a niños víctimas de violencia^23^, pero aún existe un vacío en el conocimiento sobre los sentimientos expresados por los profesionales que atienden a víctimas de violencia sexual infantojuvenil.

La relevancia de este estudio radica en la posibilidad de contribuir a mejorar la preparación emocional de los profesionales que trabajan en los servicios de salud de cualquier nivel asistencial. Esta medida ayuda a poner en práctica las recomendaciones del Ministerio de Salud para que estos trabajadores estén preparados y se impliquen en la atención a las víctimas y sus familias^4^. Sin embargo, también sirve para subsidiar las políticas de salud ocupacional en los servicios de salud y, consecuentemente, extender el alcance de estas políticas a los diversos contextos en que infantojuveniles son atendidos, como escuelas, guarderías, Consejos Tutelares y Comisarías Especializadas, entre otros, ya que los sentimientos expresados pueden extenderse a personas que interactúan y conviven en una misma comunidad. El objetivo de este estudio es describir los sentimientos expresados por profesionales de la salud al atender a infantojuveniles víctimas de violencia sexual a partir de la perspectiva teórica del Interaccionismo Simbólico.

## Método

### Tipo de estudio

Estudio cualitativo presentado según la herramienta *Consolidated Criteria for Reporting Qualitative Research* (COREQ) adaptada para Brasil^24^, producto de una tesis de maestría defendida en 2020 en un programa de posgrado del país.

### Local del estudio

La investigación tuvo lugar en un hospital público del nordeste de Brasil que presta asistencia general, ambulatoria, clínica, quirúrgica y de urgencia/emergencia a todos los grupos de edad y a ambos sexos. El campo se abordó a través del trabajo de la primera autora como enfermera en el hospital. Se visitaron las unidades de cuidados abiertos (clínicas) y cerrados (unidades de cuidados críticos) para pacientes pediátricos y adolescentes. El proyecto se presentó al consejo de administración, a los responsables de las unidades asistenciales y a los posibles participantes en una reunión previa.

### Participantes del estudio

Para seleccionar a los participantes, se abordó individualmente a todos los profesionales de salud que se encontraban en las unidades de atención y se los invitó a participar en la investigación. Tras la aceptación, se verificaron los criterios de inclusión: trabajar más de un año en la institución y haber atendido a niños y adolescentes víctimas de violencia sexual; y los criterios de exclusión: estar ausente del servicio de investigación por cualquier motivo y trabajar en unidades de apoyo al diagnóstico.

No hubo negativas ni retiradas por parte de los invitados a participar en la investigación. Como resultado, de los 36 profesionales contactados aleatoriamente, sólo 31 cumplían los criterios de inclusión, pero un entrevistado fue descartado por falta de información.

### Recopilación de datos

La recogida de datos tuvo lugar en junio y julio de 2019, realizada por la estudiante de máster en compañía de cuatro estudiantes de enfermería (todas ellas mujeres) que participaron individualmente en la entrevista para comprobar el cumplimiento del guion previsto. Todas las estudiantes estaban vinculadas a un grupo de investigación, debidamente cualificado para la recogida de datos cualitativos en un curso de extensión frecuentado en el mismo año.

Inicialmente, se aplicó un cuestionario estructurado y un guión de entrevista semiestructurada previamente creado por la investigadora, que incluía la siguiente pregunta: ¿cómo se sintió al asistir a las víctimas? Sin embargo, a lo largo de los informes, los profesionales también mencionaron las reacciones observadas en otros profesionales durante el proceso de asistencia a las víctimas. 

Las entrevistas en profundidad se concertaron previamente con los participantes. Los diálogos, que tuvieron lugar una sola vez durante las horas de trabajo de los profesionales (de día y de noche) y duraron una media de 45 min, fueron grabados en audio. Cabe destacar que la saturación temática de los datos se identificó con el participante número 23 y se confirmó con otras siete entrevistas que eran similares en cuanto a las experiencias compartidas, respondían al objetivo propuesto y no aportaban nada a la interpretación y el análisis de los datos.

### Análisis de datos

Una vez recogidos los datos, los estudiantes los transcribieron, pero sólo una participante accedió a leer y corregir su entrevista transcrita. Ante esta dificultad, la corrección y validación textual de las demás participantes corrió a cargo de la investigadora responsable. Se utilizó la técnica de análisis temático de contenido sugerida por Bardin^25^ en tres etapas: a) preanálisis; b) exploración del material; c) tratamiento de los resultados e inferencia.

En la etapa de preanálisis, se transcribieron las entrevistas, se imprimieron y se leyeron superficialmente en su totalidad. A continuación, se extrajeron temas preliminares que suscitaron la mención de sentimientos expresados que también estaban presentes en las observaciones de los investigadores, en las conversaciones posteriores a cada entrevista y que se anotaron en las entradas del diario reflexivo. 

La segunda fase -exploración del material- comenzó manualmente. Un grupo de reflexión se reunió semanalmente para analizar, debatir, reflexionar y compartir impresiones e interpretaciones de los datos. Los textos transcritos se releyeron constantemente (cada entrevista fue leída al menos ocho veces por el investigador principal y tres veces por los académicos). En este proceso, las lecturas en profundidad se realizaron al mismo tiempo que la escucha repetida (al menos cinco veces) de cada entrevista, con el fin de permitir que los textos se impregnasen de los investigadores. Tras este proceso, los textos se importaron al *software* NVivo versión 12, donde se organizaron y sistematizaron en códigos, o nodos temáticos.

Para proceder a la tercera etapa - interpretación - las declaraciones de los participantes se agruparon en códigos basados en la información convergente, dando lugar a un total de 23 expresiones y términos que se consideraron subtemas o subcategorías, agrupados por significado convergente en categorías temáticas^25^. La validación del proceso de análisis se basó en discusiones con tres académicos de enfermería y tres investigadores doctores, así como en un momento privado con cinco participantes en la investigación que confirmaron los resultados presentados. El proceso de análisis de datos se describe en la Figura 1. 

**Figura 1:** Ejemplos de expresiones que dieron lugar a categorías temáticas desde la perspectiva del Interaccionismo Simbólico. Salvador, BA, Brasil, 2019

Objeto simbólico	Expresiones mostradas en las interacciones sociales	Significado	Significante
Niño/adolescente agredido sexualmente	“*podrían ser sus hijos*”	Reflexionar sobre su posible sufrimiento al vivir una situación similar (que violen a su hija/adolescente) y sopesar el sufrimiento que puede estar experimentando la otra persona al violar a su hija.	Empatía
	“*piensa en el sufrimiento de la mujer con su hija en este estado*”		
	“*me pongo en el lugar de la mujer*”		
Acción de buscar información para elucidar la sospecha de violencia sexual	“*miedo de hacer demasiadas preguntas y que la madre se queje*”	Posibles reacciones de las familias de las víctimas a las preguntas sobre sospechas de violencia sexual.	Miedo
	“*miedo a decir sospechas ... la familia amenaza*”		
Acciones y reacciones del agresor ante la violencia sexual contra niños/adolescentes	“*indignado*”	La reacción ante una agresión sexual contra niños y adolescentes es rechazar al agresor. La opinión converge en la necesidad de insurgencia e intolerancia por parte de la sociedad contra este acto y contra quienes no expresan el mismo sentimiento	Indignación
	“*me molestó ... mamá no mostró enfado*”		
Presentación de un cuadro clínico complejo que requiere esfuerzo físico y emocional por parte de los profesionales	“*el doctor lloró mucho [...] me sentí fatal*”	Un acto reflejo al darse cuenta de que la víctima también sufre, o incluso el resultado de los esfuerzos físicos y emocionales realizados durante la asistencia.	Sufrimiento
	“*como si me hubieran chupado la energía*”		
Largo período de tiempo después de recibir atención	“*hasta el día de hoy, permanece en mi mente [...] todo el mundo está emocionalmente triste*”	Una reacción de profundo desánimo ante alguna situación movilizadora; esta reacción puede durar independientemente de la pérdida (y/o distanciamiento físico) o incluso del distanciamiento emocional de un objeto social, en estos casos, la historia de la víctima.	Consternación
	“*me lleva un tiempo procesar estas historias [...] no puedes evitar sentirte movilizado y triste*”		

Los análisis, inferencias e interpretaciones recomendados por Bardin^25^ están anclados en los fundamentos filosóficos del Interaccionismo Simbólico (IS), que se encuentran en “Mente, Yo y Sociedad” de Charles Morris. El precursor de esta teoría fue el científico social George Herbert Mead, cuyas ideas fueron sistematizadas por su asesor doctoral Herbert Blumer. Durante el siglo XIX, la SI se incorporó a la psicología social, dando lugar a un marco teórico que consta de tres premisas básicas: 1) la forma en que una persona interpreta los hechos y actúa depende del significado que se les atribuye; 2) el significado se construye a través de procesos interactivos en la sociedad; y 3) con el tiempo, los significados pueden sufrir cambios^20^.

Esta teoría se centra en las interacciones sociales, las respuestas humanas y la influencia que la inmersión de un individuo en la sociedad tiene en sus actitudes hacia las situaciones que experimenta^20^. Así, se identificaron cinco categorías temáticas: empatía, miedo, indignación, sufrimiento y consternación.

### Aspectos éticos

El proyecto fue aprobado por el Comité de Ética de la Investigación y respeta las Resoluciones del Consejo Nacional de Salud de Brasil (466/2012 y 510/2016). De esta forma, todas las personas que participaron en la investigación, tras leer y dar su conformidad, firmaron las dos copias del Formulario de Consentimiento Libre e Informado. 

Las entrevistas se realizaron en salas privadas situadas en la institución de investigación, en presencia de la investigadora principal y de una estudiante de enfermería, a fin de garantizar la confidencialidad y el secreto de la información facilitada. Para preservar el anonimato de los participantes, sus nombres se han sustituido por “P” (en referencia a profesional), seguido de un número arábigo, según el orden en que se realizaron las entrevistas: P1, P2, P3 ... P30.

## Resultados

Participaron 30 profesionales de salud, de los cuales el 33,3% eran enfermeros, el 33,3% técnicos de enfermería, el 10,0% trabajadores sociales, el 10,0% médicos, el 6,7% auxiliares de enfermería y el 6,7% psicólogos. Todos los participantes declararon ser mujeres, autodeclararon una identidad de género cis femenina, orientación sexual heterosexual, un 80% de raza/color negro y una edad comprendida entre los 25 y los 65 años. Además, el 73,3% había completado más de diez años de formación profesional, el 70% había trabajado en el sector de salud durante más de diez años y el 40% había trabajado en el hospital donde se realizó la investigación durante más de diez años.

Tras el proceso analítico, surgieron cinco categorías temáticas que representan los sentimientos expresados por los profesionales y evidenciados en los informes, a saber: empatía, miedo, indignación, sufrimiento y consternación, como se describe a continuación.

### Empatía

Según los informes de los participantes, el sentimiento más evocado fue la empatía. La empatía significa tener la capacidad de ponerse en el lugar del otro sin pasar necesariamente por las mismas experiencias que él. La empatía se considera un sentimiento relevante e indispensable en los cuidados, como se ha mencionado anteriormente:

*La agresión sexual a un niño o adolescente interfiere mucho en tus emociones. Inmediatamente piensas que podrían ser tus hijos* (P11, Técnico de enfermería).

*Se te acelera el corazón. Vemos la situación de este ser humano, de este niño/adolescente. No sé cómo alguien puede llevarse a un niño, a un bebé, como ya ha ocurrido. Me pregunto: ¿qué les está pasando a los seres humanos?* [...] (P26, Trabajadora social).

*Las mujeres son víctimas de violaciones a gran escala. Esto afecta a nuestro lugar como mujeres, que estamos subyugadas. Pensamos en el sufrimiento de esta mujer, madre, abuela cuando ve a su hija en este estado* (P12, Trabajadora social).

*Me pongo mucho en el lugar de la mujer. Para mí, sería lo peor que podría sufrir en mi vida.* [...] (P13, Enfermera).

### Miedo

El miedo fue otro sentimiento expresado por los profesionales del estudio, que ocurrió principalmente durante la recogida de información sobre el caso de violencia sexual. Esta información se recoge en los servicios de salud a partir de los informes de los responsables de las víctimas, pruebas de laboratorio, pruebas de imagen y anamnesis. El miedo también se producía explícitamente durante el acto de comunicar el diagnóstico (o incluso la sospecha de este) a los familiares de las víctimas:

[...] *la zona sangraba y estaba muy abierta. Teníamos miedo de hacer demasiadas preguntas y de que la madre se quejara a la dirección del hospital de que estábamos haciendo demasiadas preguntas, ¡lo que la avergonzaba!* (P1, Técnico de enfermería)*.*

*Teníamos miedo de decir que había sospechas de violencia sexual porque la familia suele amenazar al equipo* (P19, Técnico de enfermería)*.*

*Cuando la otra técnica y yo fuimos a cambiarle el pañal y a hacerle la higiene íntima, nos dimos cuenta de que tenía el ano muy dilatado.* [...] *teníamos miedo de decir algo sobre la sospecha y el padre reaccionaría mal* (P10, Técnico de enfermería)*.*

### Indignación

Los colaboradores manifiestan indignación, revuelta y desesperación cuando tratan con víctimas de violencia sexual, ya sean niños o adolescentes. Sus sentimientos son tan fuertes que, en algunos casos, se rebelan contra el agresor:

[...] *un niño de seis meses, llorón y asustado. El médico encontró fisuras, queloides y hemorragias en el recto. El anestesista y yo estábamos muy indignados* (P2, Auxiliar de enfermería)*.*

[...] *el familiar le enseñó los genitales, la amenazó* [a la adolescente] *de muerte si se lo contaba a sus padres. ¡Era absurdo! ¡Estaba deseando que muriera el agresor!* (P14, Enfermera)*.*

[...] *era una chica inocente que ni siquiera sabía lo que le había pasado. Tenía los genitales lacerados. Lo que más me molestó fue la madre, que en ningún momento mostró enfado* (P9, Técnico de enfermería)*.*

*¡El anestesista* [médico] *estaba indignado! Todo el equipo estaba indignado* (P8, Técnico de enfermería)*.*

### Sufrimiento

Los profesionales entrevistados relataron que se sentían tan afectados por los casos de violencia sexual contra niños y adolescentes que varios miembros del equipo dijeron que el llanto era la principal expresión de la emoción que experimentaban (una mezcla de rabia, tristeza, ira y empatía con la víctima). El sufrimiento de las víctimas también reflejaba y generaba sufrimiento en los profesionales durante su asistencia:

*El niño tenía unos dos años.* [...] *Estaba herido desde los genitales hasta el año. El médico lloraba mucho mientras le operaba. Me sentí fatal* (P9, Técnico de enfermería)*.*

*El cirujano y yo estábamos muy conmocionados, porque era un niño de ocho meses que llegó en un estado muy grave, con lesiones importantes en el ano y la vagina. Yo estaba muy afectada emocionalmente* (P25, Médico)*.*

[...] *son casos difíciles que implican nuestros sentimientos. Después del servicio, me siento agotada, como si me hubieran chupado la energía* (P27, Psicóloga)*.*

### Consternación

La imagen de las víctimas y los recuerdos de sus historias están presentes de forma recurrente en la memoria de los profesionales, despertando sentimientos de consternación. Según los participantes, esto ocurría incluso después de haber pasado mucho tiempo atendiendo a estos niños y/o adolescentes:

*Ocurrió hace un par de años y, hoy, todavía se me queda grabado cuando ese niño llegó con la región perianal sangrando y pasó por el quirófano* [...] *hasta hoy día, todos estamos emocionalmente tristes* (P1, Técnico de enfermería)*.*

*Atendí a un niño que tenía menos de un mes y murió.* [...] *en casa sólo podía pensar en eso. Durante mucho tiempo, cuando trabajaba, miraba la cama y sólo podía pensar en ella* [...] *y todo el mundo está muy triste por este recuerdo* (P23, Auxiliar de enfermería)*.*

*La madre llevó a la niña a urgencias porque sospechaba que el agresor era un familiar.* [...] *cuando trato con casos de abuso sexual de niños y adolescentes, me lleva un tiempo procesar estas historias porque no puedes evitar sentirte muy movilizada y triste después de tratar un caso como este* (P12, Trabajadora social)*.*

## Discusión

De acuerdo con los relatos de los participantes, los sentimientos más frecuentemente evocados fueron: empatía por las víctimas; miedo de denunciar sospechas de violencia sexual, de orientar la atención hacia ese problema y de ser objeto de represalias; indignación ante el hecho ocurrido (violencia sexual contra niños y adolescentes) y ante la inercia inesperada de la familia de la víctima; sufrimiento ante los casos clínicos y las situaciones asistenciales vividas; consternación ante la víctima, los hechos denunciados y la atención prestada.

Estos hallazgos ponen de manifiesto la complejidad del individuo implicado en la atención a víctimas infantojuveniles de violencia sexual. Aunque la VSI esté instalada en la sociedad y esto se evidencie en diversos estudios^7^ ^)-(^ ^12^, su ocurrencia en la vida real aún moviliza las emociones de los profesionales que trabajan con ella, hasta el punto de hacer aflorar sentimientos que pueden llevarlos a la parálisis total o a iniciar las acciones asistenciales necesarias, proceso que depende de la percepción del mundo de cada persona. 

Según Morris, la percepción es una especie de mediador necesario para las interacciones entre las personas^20^. Desde esta perspectiva, la prestación de cuidados requiere empatía, que posibilita la creación de lazos de afecto, comprensión y reciprocidad afectiva. Esto se debe a que, al utilizar la empatía para brindar atención a víctimas infantojuveniles de violencia sexual, los profesionales de la salud involucran sentimientos de fraternidad y compasión, considerados importantes para el cuidado humano^21^. La empatía es un sentimiento que da a un individuo la capacidad psicológica de sentir lo que otra persona sentiría si estuviera en la misma situación^19^ ^)-(^ ^21^, lo que ocurre a través de interacciones.

Estudios nacionales e internacionales sobre la vivencia de violencia doméstica en la infancia y/o adolescencia muestran que, independientemente del tipo de violencia y del número de ocurrencias, las víctimas sufrieron daños físicos, emocionales y psicológicos. En muchos casos, estos problemas resultan en diversas complicaciones clínicas, sufrimiento intenso y la consecuente necesidad de atención especializada, que también afecta a sus familias y alcanza el estatus intergeneracional^7^ ^),(^ ^11^ ^),(^ ^22^ ^),(^ ^26^ ^)-(^ ^27^. 

Se puede afirmar, por tanto, que la violencia sexual causa sufrimiento a las víctimas, a sus familias y al equipo de salud. En este sentido, la interacción profesional-paciente es necesaria para la atención y debe estar mediada por la empatía, una herramienta importante para acercarse y acoger a las víctimas y sus familias^20^.

Corroborando la discusión, estudiosos^25^ afirman que es posible inferir que la interpretación de las vivencias de los niños víctimas puede ser resignificada en la edad adulta. Esta posibilidad no excluye a los entrevistados, es decir, dependiendo de la historia de vida personal de cada profesional y de cómo vivieron (o no) situaciones de violencia en la infancia/adolescencia, los significados atribuidos a su papel de cuidadores pueden verse comprometidos^12^ ^),(^ ^20^ ^),(^ ^26^ ^)-(^ ^27^, lo que podría generar temor.

El miedo a las represalias es un sentimiento común entre los profesionales que atienden a víctimas de violencia sexual. Este miedo puede verse exacerbado por la inseguridad, el agotamiento físico y emocional, la falta de apoyo adecuado para llevar a cabo las actividades asistenciales y el estrés, que son problemas laborales comunes en los servicios de salud nacionales e internacionales^17^ ^),(^ ^23^ ^),(^ ^28^ ^)-(^ ^29^. Hay que tener en cuenta que la violencia sexual infantil y adolescente puede hacer que los familiares se sientan tan desesperados y emocionalmente fuera de control que se vuelvan contra los profesionales durante la atención, como se ha mencionado anteriormente.

Es cierto que en contextos violentos, la sociedad en su conjunto^20^, incluidos los que se ocupan de atender a estas víctimas, se ve sacudida. Por ejemplo, en Atención Primaria de Salud, los profesionales se desplazan y se integran en la comunidad ante la necesidad de atender diversos problemas a través de estrategias como la notificación en el Sistema de Información de Enfermedades de Declaración Obligatoria (SINAN, por su acrónimo en portugués). En este sentido, un estudio realizado en Recife, Pernambuco, revela que la sensación de amenaza puede desencadenar un sentimiento de miedo, que se considera un impedimento para la notificación obligatoria de la violencia contra niños y adolescentes, aunque los profesionales lo reconozcan como una obligación ética y legal^30^. 

Sin embargo, un estudio nacional realizado con 242 trabajadores de enfermería revela que 20% y 59% de esos profesionales relatan haber sufrido agresiones físicas y verbales, respectivamente, durante el horario de trabajo^31^, experiencia compartida por enfermeros de dos Unidades de Atención de Urgencia de Paraná^32^. En el ámbito internacional, un estudio realizado en Suecia con 1.567 profesionales de la salud encontró una prevalencia de casi 25% de experimentar violencia en el lugar de trabajo^33^, una realidad similar a la vivida por 769 médicos noruegos, que informaron una prevalencia de 20,3% de amenazas múltiples de violencia en los primeros cuatro años de carrera profesional^29^.

La edad media de los participantes, 40 años, y los más de 10 años de experiencia profesional en el sector de salud fueron elementos importantes a la hora de analizar estos resultados. En comparación, un estudio realizado en Noruega afirma que ser más joven es un factor asociado a que los profesionales experimenten violencia física en su trabajo en el área de la salud^29^, realidad que difiere de la de este estudio. Sin embargo, se puede confirmar la hipótesis de que las angustias de los profesionales eran reales, dado que el miedo a las represalias y a las posibles reacciones agresivas de los familiares de las víctimas no es un sentimiento infundado.

Por otro lado, es posible que haya que superar este miedo durante el trabajo profesional para poder prestar una atención eficaz. Un estudio revela que existen mecanismos de “construcción de sentido” utilizados por los supervivientes de abuso sexual infantil, como movilizar recursos sociales y contar con el apoyo de la sociedad^34^. Estos datos apuntan a la necesidad de que los profesionales de la salud sean capaces de actuar como apoyo social para las víctimas infantojuveniles, incluyendo la superación del miedo antes mencionado.

Además, la confianza mutua que se establece entre los profesionales es responsable de aumentar la percepción de seguridad y mejorar el control emocional del equipo. De esta forma, es posible reducir el estrés y aumentar la confianza mutua en un ciclo de refuerzo positivo^28^. Sin embargo, la falta de tiempo y la ausencia de instrumentos legales que regulen las atribuciones de los profesionales brasileños, especialmente de enfermería, son desafíos impuestos al cuidado de las víctimas de violencia sexual^17^ y pueden, como consecuencia, contribuir a aumentar la sensación de inseguridad durante la asistencia.

Aunque no fue el resultado de este estudio, el miedo a la responsabilidad legal en relación con los procesos judiciales que se pueden iniciar tras una denuncia de violencia sexual es también un sentimiento que afecta a las enfermeras que atienden a las víctimas^4^ ^),(^ ^17^. Talvez esto se deba a la falta de instrumentos legales que regulen sus atribuciones y su papel en la cadena de custodia de las pruebas^17^, lo que está relacionado con la falta de un enfoque temático durante la formación y anima a los futuros profesionales a actuar en primera línea en la atención a las personas que han sufrido violaciones sexuales^35^.

Además de la formación académica, dependiendo de la interacción social de estos profesionales, el simbolismo atribuido a la violencia sexual y cómo estos profesionales muestran sus sentimientos, la forma en que reaccionan a la necesidad de atender a la víctima puede ser matizada. Para interaccionistas como Morris, las experiencias sociales influyen en las reacciones de las personas y pueden ser responsables (junto con la construcción social) de las acciones de los individuos ante las situaciones que viven^20^. Así, el miedo relatado puede hacer que los profesionales no se sientan preparados para actuar en la atención a las víctimas, dificultando procesos como la notificación y la denuncia, aunque muestren indignación cuando identifican este tipo de agresiones contra niños y adolescentes. Posiblemente, esa indignación esté relacionada con la capacidad del individuo como ser social de ponerse en el lugar del otro, como revela Morris^20^, o simplemente de transferir la responsabilidad, como denuncian los profesionales que actúan en los diversos niveles de atención en Brasil^12^. De esta forma, la indignación es una respuesta sentimental y se relaciona con el crimen - significado atribuido a este tipo de violencia por los profesionales de salud, aunque no sea el motor de las acciones asistenciales.

Por lo tanto, se puede inferir que las reacciones a la atención prestada a las víctimas provocaron un intenso sufrimiento entre los profesionales, además de contribuir a que los hechos se tornaran inolvidables, incluso cuando ocurrieron hace muchos años. Además, los desencadenantes emocionales provocados por los recuerdos vividos pueden ser responsables de la consternación antes mencionada.

De hecho, la atención a víctimas de violencia sexual fue señalada como una situación que marca y conmociona a todo el equipo. Aunque difícil, la actuación del profesional se basa en prerrogativas legales (como están establecidas en los códigos de ética y en el ECA) e incluye la notificación y la denuncia de los casos sospechados/identificados, acciones que, si son desatendidas, generan sanciones como multas^3^. Estos avances en la asistencia de salud brasileña definen la actuación de los profesionales en la atención a las víctimas, una situación totalmente sistematizada y con directrices explícitas^4^, si se compara con la situación opuesta, como la organización y el flujo de registros en el sistema de salud de Arabia Saudí^13^.

El desarrollo de procesos formativos dirigidos a capacitar a los profesionales para gestionar el proceso de atención a estos casos es una piedra angular en la preparación emocional de los profesionales. Las investigaciones confirman que la cualificación de los profesionales mediante la inclusión de contenidos en su formación académica aumenta la posibilidad de que implementen políticas públicas preexistentes^17^ ^),(^ ^35^, aunque no fue así en un estudio asiático con 300 médicos^13^. Por otro lado, el uso de herramientas interactivas promueve la atención segura de las víctimas de violencia sexual, reduce el sentimiento de impotencia del profesional y apoya el razonamiento clínico necesario para llevar a cabo el proceso de atención médica^18^. 

Teniendo en cuenta estos resultados, es plausible que los responsables del programa de salud ocupacional en las instituciones necesiten investigar los signos y síntomas relacionados con el estado mental de los profesionales que trabajan en los servicios para víctimas de violencia sexual, considerando los impactos negativos mencionados anteriormente. Además, estos profesionales necesitan intervenciones continuas a corto, medio y largo plazo para promover acciones que reduzcan el riesgo de desarrollar trastornos mentales leves, moderados y más graves.

Se recomienda la realización de nuevos estudios sobre este tema utilizando otras metodologías. También se sugiere que este estudio sea replicado en otros contextos como la educación (guarderías, escuelas), los servicios de salud (atención primaria, servicios especializados y maternidades), los servicios de seguridad pública, protección de niños y adolescentes y justicia (como el Consejo Tutelar, las Comisarías Especializadas, el Ministerio Público y la Defensoría Pública), así como con otros grupos sociales (profesionales de la seguridad pública, profesores y cuidadores informales) para comparar los resultados aquí obtenidos y verificar similitudes y/o diferencias. 

Este estudio se limita a revelar los sentimientos de mujeres profesionales que trabajan en un servicio de salud hospitalario. Sin embargo, estos resultados están abiertos a la generalización, ya que se trata de un tema que provoca una movilización emocional global en las sociedades occidentales, organizadas moralmente a la luz de las conductas que orientan la protección y los derechos de los niños y adolescentes. Además, el estudio está abierto a la replicación, ya que utilizó la teoría social y el rigor metodológico para garantizar la fiabilidad de los resultados.

## Conclusión

Empatía, miedo, indignación, sufrimiento y consternación son sentimientos expresados por el equipo cuando atiende a víctimas de violencia sexual infantojuvenil. El énfasis en estos sentimientos hace que la experiencia de atender a estas víctimas sea difícil y notable, movilizando sentimientos que pueden interferir en la eficacia de la atención prestada.

Este estudio refleja la realidad de los profesionales que prestan atención a las víctimas de violencia sexual, por lo que recomendamos su uso para cualificar a los profesionales que pueden hacer frente a situaciones similares. Además, el estudio podría orientar la formación de salud sobre el tema y motivar la implementación de políticas públicas actuales, como la prestación de asesoramiento psicológico al equipo de salud responsable de la atención a las víctimas, tal y como recomienda el Ministerio de Salud.
